# Developing a tailored bio-behavioural survey on viral hepatitis among migrants: mixed-methods preparations for the HepMig pilot study, Germany, July 2022–March 2023

**DOI:** 10.1186/s13104-025-07516-5

**Published:** 2025-10-20

**Authors:** Anna-Lisa Behnke, Ida Sperle, Gyde Steffen, Navina Sarma, Anastassiya Stepanovich-Falke, Sandra Dudareva, Ruth Zimmermann, the HepMig Study Group, Leman Bilgic, Münevver Demir, Claudia Hövener, Carmen Koschollek, Anna Kühne, Majdi Laktinah, Timo Ulrichs, Janka Vogel

**Affiliations:** 1 Department of Infectious Disease Epidemiology, Robert Koch Institute, Berlin, Germany; 2 Postgraduate Training for Applied Epidemiology (PAE), Department of Infectious Disease Epidemiology, Robert Koch Institute, Berlin, Germany; 3 ECDC Fellowship Programme, Field Epidemiology Path (EPIET), European Centre for Disease Prevention and Control (ECDC), Stockholm, Sweden

**Keywords:** Integrated bio-behavioural survey, Hepatitis B, Hepatitis C, Hepatitis D, Migration, Health surveys, Study design, Sampling, Data collection, Multilingual, Community participation

## Abstract

**Objective:**

Migrants in Germany from countries with a high prevalence of viral hepatitis may need improved access to healthcare services but have not been adequately included in health surveys so far. The *HepMig* pilot study aims to develop and pilot an integrated bio-behavioural survey on viral hepatitis among migrants from high-prevalence countries living in Germany. The design should be robust, acceptable, feasible, non-stigmatising, non-discriminatory, and community-centred. This paper describes the preparatory research that informed the pilot study design.

**Results:**

We defined our populations of interest as adults (≥ 18 years) born in Bulgaria, Romania, Russia, Syria, or Türkiye living in Germany, irrespective of current citizenship or residence status. Analysis of sociodemographic data from the Federal Statistical Office (DESTATIS) revealed considerable differences between these populations in age distribution, duration of residence, and geographic distribution in Germany. An inductive analysis of information provided by community experts – who shared the same country of birth and /or history of migration or closely worked with the populations in focus – from clinical medicine, academia, non-governmental organizations, and public health identified four themes: (i) sampling of heterogeneous populations of interest; (ii) accessibility and trustworthiness as prerequisites for study participation; (iii) opportunities for community engagement and empowerment; and (iv) ethical considerations. Results will inform sampling, recruitment, data collection, and piloting the study design.

**Supplementary Information:**

The online version contains supplementary material available at 10.1186/s13104-025-07516-5.

## Introduction

Germany aims to eliminate viral hepatitis as a public health threat by 2030 [1]. Targeted prevention and treatment services are needed, based on reliable and in-depth data about the prevalence of infections with hepatitis B and C viruses (HBV; HCV), risk and protective factors, and access to healthcare services. A nationwide representative serosurvey found low prevalences of 0.3% for HBV and 0.2% for HCV in the general population [2]. There is a higher prevalence in sub-populations that include migrants from high-prevalence countries [3–5]. A study estimated that 55% of chronic HBV and 25% of chronic HCV infections in Germany are attributable to first-generation migrants born in high-prevalence countries [6]. However, large representative studies on HBV and HCV burden and access to healthcare in these populations are missing. Previous studies either failed to include all migrant populations, focussed solely on refugees [7–9], or did not define or assess relevant migration- and healthcare-specific information.

Including migrants in health studies can be challenging due to a variety of barriers (e.g. sociodemographic, language, structural, practical, and emotional) [10, 11]. While efforts have been made to improve the inclusion of migrants in the national health monitoring [10, 12], studies with a focus on viral hepatitis are still missing. Given the stigma around hepatitis B and C [13, 14], approaches must be non-stigmatizing and appealing to the target population [10, 11].

The HepMig pilot study was initiated to develop and pilot a robust, acceptable, feasible, and non-stigmatizing study design for an integrated bio-behavioural survey on viral hepatitis among migrants from high-prevalence countries living in Germany. In this paper, we describe the mixed-methods preparatory steps to define and describe our populations of interest and gather input from community experts to develop appropriate and well-accepted sampling and recruitment methods. As part of this study, we further piloted the study design in two migrant populations in one German city and evaluated its feasibility and acceptability (publication of pilot study results in preparation). Results from mixed-methods preparatory steps and pilot study will inform a nationwide study that is planned for the future. Findings will be useful to other researchers facing comparable challenges.

## Main text

### Methods

We chose a mixed-methods design for the preparatory steps, consisting of qualitative and quantitative methodological parts.

To define our populations of interest, we first operationalized the relevant ‘migration status’ of a person as being foreign-born and living in Germany, irrespective of current citizenship and residence status [15]. To select populations of interest by country of origin, we secondly updated the estimated numbers of HBV- and HCV-infected people living in Germany in 2020 using a modified workbook method [16]. This technique combines population size with prevalence data and/or estimates in selected groups, in our case first-generation migrants with non-German citizenship (as a proxy for country of origin) of a high-prevalence country (details described elsewhere [6]). For practical reasons and mindful of limited resources, we selected the five populations with the highest numbers of HBV- and HCV-infected people with non-German citizenship living in Germany as our populations of interest.

To map the sociodemographic distribution of the selected populations of interest, we described them by mean age, proportions of age groups, duration of residence, and geographic distribution in Germany. We used two federal statistical data sources: (1) for age, age groups, and duration of residence we used the ‘Mikrozensus’, that provides information on foreign-born citizens with a statically defined, so-called migration background [17]; (2) for geographic distribution on municipal level we used the ‘Ausländerzentralregister’, that contains information on foreigners with non-German citizenship [18] (data as of December 31, 2021).

To gain insights into potential sampling and recruitment methods, we conducted key informant interviews with community experts from academia, clinical medicine, non-governmental organizations, and public health. The community experts shared the same country of birth and/or history of migration as the study population or closely worked with the populations in focus.

 Interviews either had a focus on the selected populations of interests or on overarching healthcare aspects. We used a semi-structured interview guide that covered topics such as potential community partners and locations for recruitment, appropriate data collection methods, and needs with regards to study materials (see Supplement 1). Interviews were conducted by HepMig study team members using phone or videocall. The duration of interviews ranged from 20 to 90 min. Interviews were documented in a word template. One commissioned expert per selected country of birth conducted a situation analysis covering topics such as proportion and distribution of people living in Germany not registered, reachability of sub-groups, and relevant community networks.

To further discuss sampling and recruitment methods, we invited selected experts to a workshop with moderated thematic discussions and country-specific working groups. We created an interdisciplinary advisory board with experts – the majority with a history of migration from the selected countries – from academia, clinical medicine, non-governmental organizations, and public health, for continuous critical review of proposed methods.

The first author analysed the qualitative data (interviews and meeting notes) through an inductive approach. Identified themes and topics were discussed and revised among the research team to be considered in study design development.

### Results

#### Selection of populations of interest

Based on estimated numbers of HBV- and HCV-infected people with non-German citizenship living in Germany in 2020 (Table 1), we selected Bulgaria, Romania, Russia, Syria, and Türkiye as relevant high-prevalence countries [5] of birth for our populations of interest.

**Table 1:** Estimated numbers of adults ( >= 18 years) with non-German citizenship living in Germany in 2020 [18, 19] positive for hepatitis B surface antigen (HBsAg) [4, 20] and/or HCV ribonucleic acid (HCV RNA) [21]

Country	Estimated number of adults positive for HBsAg	Estimated number of adults positive for HCV RNA
Türkiye	37,599	2,820
Romania	33,951	14,996
Syria	12,585	7,205
Bulgaria	10,832	3,611
Russia	6,260	5,800
Italy	6,051	5,843
Poland	6,660	3,297
Vietnam	8,438	784
Ukraine*	1,715	3,786

HBsAg: hepatitis B surface antigen; HCV RNA: hepatitis C virus ribonucleic acid

*The size of the Ukraine-born population increased considerably after March 2022 and might impact the selection for the main study

#### Sociodemographic mapping of populations of interest

**Fig. 1:**
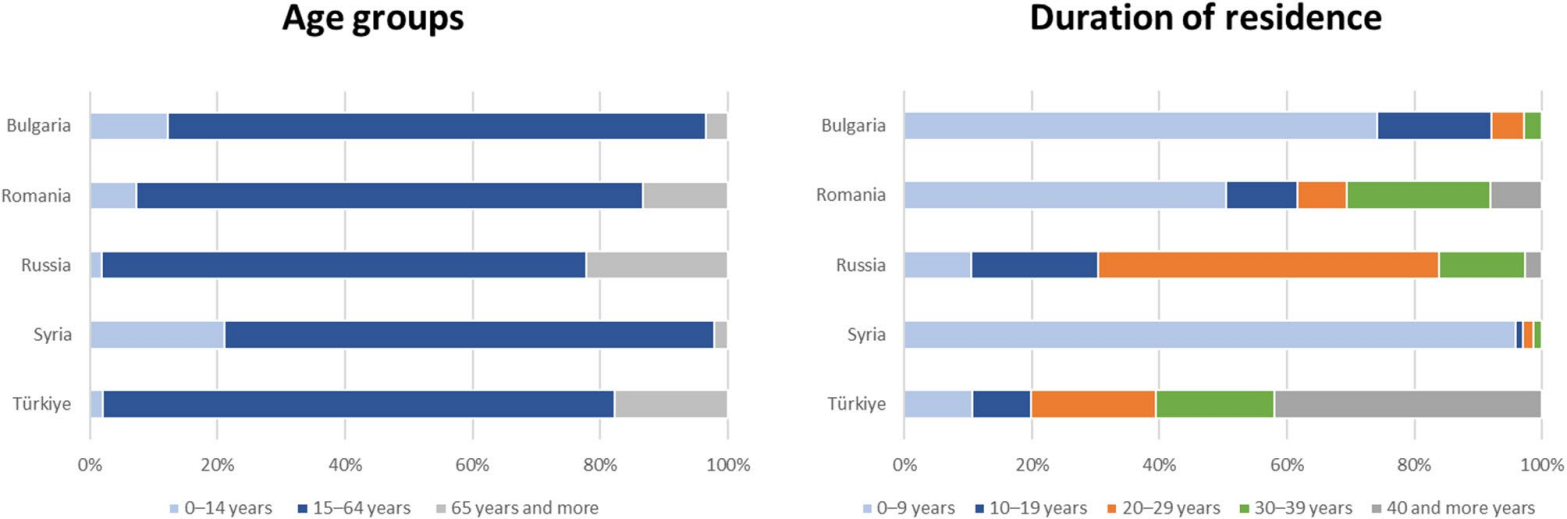
Proportions of people born in Bulgaria, Romania, Russia, Syria, and Türkiye by age groups (left) and duration of residence in Germany (right), Germany, 2021. From: DESTATIS, Mikrozensus 2021 [17]

As of December 31, 2021, a total of 4,223,000 people were born in Bulgaria, Romania, Russia, Syria, or Türkiye and registered in Germany, which corresponds to 5.2% of the German population and 30% of the foreign-born population in Germany [17]. Age distribution and duration of residence in Germany of people born in the selected countries differed (Fig. 1). The mean age of migrants born in Türkiye was 51 years and in Syria 29 years [17]. The mean number of years spent in Germany was 33 years for migrants born in Türkiye and 7 years for migrants born in Syria [17].

**Fig. 2:**
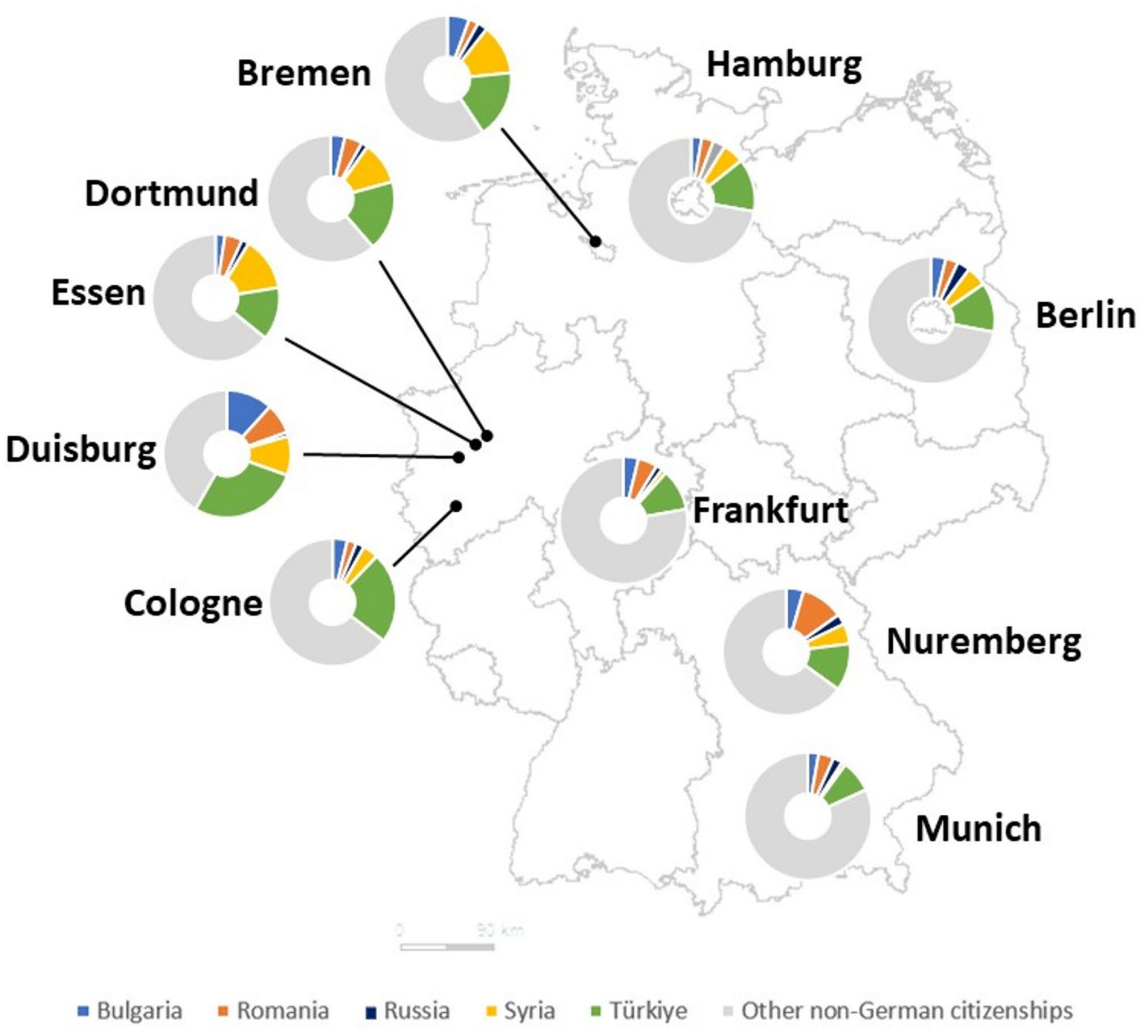
Proportions of residents with selected citizenships among all residents with non-German citizenship by major cities, as percentages, Germany, 2021. From: DESTATIS, Central Foreigners’s Registry 2021 [18]. See Supplement 2 for additional information

Analysis of geographic distribution by administrative district of residence [18] resulted in a list of cities with the highest numbers of residents with Bulgarian, Romanian, Russian, Syrian, and Turkish citizenship. Figure 2 shows a comparison of proportions of residents with the five selected citizenships among all residents with non-German citizenship for ten short-listed cities.

#### Inductive analysis of discussions with community experts

We included 29 key informant interviews (July–November 2022), five commissioned situation analyses (October 2022), discussions from our workshop meeting (23 external experts, November 2022), and discussions with the expert advisory board (March 2023–November 2023) in our thematic analysis. The following four themes were constructed.

##### Sampling of heterogeneous populations of interest

Populations of interest are diverse and heterogeneous, encompassing various migration histories (e.g., long-term vs. seasonal migration, occupational migration, refugees) and differences in healthcare access (e.g., health-insurance status, level of discrimination within the healthcare system). This diversity necessitates tailored approaches to sampling and recruitment to adequately capture the complexity of the population. A non-probability sampling approach via relevant settings was considered most effective to reach different sub-groups, i.e. different age groups, socioeconomic backgrounds, etc.

Medical, faith-based or cultural institutions, workplaces, supermarkets, and cafés could be suitable environments for sampling a high number and variety of individuals from populations of interest. Locations that lack privacy would be appropriate for raising awareness, but not for recruitment or data collection. By adding snowball sampling, family members and peers of participants could be included who would not be reached otherwise e.g., as they do not seek healthcare or attend faith-based, cultural or other institutions.

Simple random sampling was considered not possible due to the absence of a complete sampling frame as country of birth is not reliably recorded in German municipality-based residence registries [10]. In addition, a considerable number of people are not registered.

##### Accessibility and trustworthiness as prerequisites for study participation

Experts emphasized the importance of creating accessible and trustworthy environments for study participation. This includes considerations for privacy, language barriers, cultural sensitivity, and building trust with participants. Study materials should be prepared with input from community experts and translated into relevant languages. Study team and data collectors should be multilingual and could be supported by trusted community peers.

Suitable environments for data collection could be healthcare facilities, an easily accessible central study centre in a neutral, non-stigmatizing location, or participants’ homes (depending on the individual housing situation).

##### Opportunities for community engagement and empowerment

Community engagement and empowerment are crucial. Community members and people with lived experience should be involved throughout the research process by leveraging peer networks for recruitment, e.g., trusted peers as well as social media platforms. Communities could be motivated to collaborate by emphasizing the potential benefits of study participation, such as contributing to an improved knowledge base and transferring and multiplying knowledge about transmission risks and preventative measures regarding viral hepatitis.

##### Ethical considerations

Experts mentioned the need to prioritize participant well-being, ensure informed consent, mitigate against risks of stigma or discrimination during and after study participation, and safeguard participant privacy and confidentiality. Study participants might have experienced racism, stigmatization, and discrimination and feel stigmatized by this study. Therefore, the rationale of the study and focus on selected populations of interest has to be explained thoroughly.

Information on viral hepatitis should be available, preferably as a short video with clear messages. Study participants need to be enabled to make an informed decision whether to participate or not and need to be able to receive their HBV/HCV test results and support to seek follow-up diagnostic and therapeutic services, if needed. Study participants should be reimbursed for their time commitment. As a considerable proportion might not have valid health insurance, linking this group to care warrants special attention.

### Discussion

In this paper, we describe the mixed-methods preparatory steps to set up a robust, acceptable, feasible, and non-stigmatising integrated bio-behavioural survey on viral hepatitis among migrants from high-prevalence countries living in Germany. As populations of interest, we selected adults born in Bulgaria, Romania, Russia, Syria, and Türkiye, based on their large population sizes in official German registers and the high prevalence estimates for hepatitis B and C in their countries of birth. This selection is specific to Germany and does not mirror the global distribution of viral hepatitis burden. Analysis of available sociodemographic data revealed considerable differences in age distribution, duration of residence in Germany, and geographic distribution between populations from selected countries of birth. Experts noted the heterogeneity both between populations from selected countries of birth and within populations. Different sociodemographic statuses and health literacy levels likely influence access to healthcare and the burden of HBV and HCV infections [22]. Accessibility and trustworthiness are key for recruiting participants. Other important considerations include community engagement and empowerment, and ethics for designing and conducting the study.

For our study, we defined migration status as being foreign-born and we specified high-prevalence countries of birth for hepatitis B or C. This is in line with recommendations to refrain from using broad summary categories such as migration background [23]. Our findings about the need for tailored, multi-modal approaches extend findings from previous studies performed at RKI with the aim to include migrant people into representative national public health monitoring [10, 12, 24].

Given the stigma around hepatitis B and C, experts suggested a community-centred approach using comfortable and familiar environments to reach populations of interest. In contrast to register-based sampling and recruitment, a community-based approach would allow for more community engagement and collaboration with peers. However, in light of the heterogeneity of our populations of interest, including variations in age distribution, socioeconomic status, education levels, migration history and patterns, legal status, language proficiency, and health care needs, the selection of suitable environments is challenging and relevant sub-groups can easily be missed. Community-centred and ethical considerations need to be balanced with practical considerations related to the study design. Developing a study design that is tailored to very heterogeneous populations of interest and is, at the same time, feasible with limited resources is highly challenging.

## Limitations

Our preparatory study has several limitations. The relevance of our selection of countries of birth could change over time, as population sizes within Germany could vary due to different migration patterns and numbers of incoming refugees. The sociodemographic analysis was based on publicly available data from federal statistical databases. Given that a considerable proportion of our populations of interest might not be officially registered and federal statistical databases are insufficient regarding minorities with special legal status (e.g., Kurds, Palestinians, Sinti and Roma, and other groups), results should be interpreted with caution.

## Supplementary Information

Below is the link to the electronic supplementary material.

Supplementary Material 1.

## Data Availability

The quantitative data that support the findings of this study are openly available at https://www-genesis.destatis.de/genesis/online/data?operation=sprachwechsel%26language=en. The qualitative data are not openly available due to reasons of data security. Data are located in controlled access data storage at Robert Koch Institute.

## Abbreviations

HBV: Hepatitis B virus
HBsAg: Hepatitis B surface antigen
HCV: Hepatitis C virus
HCV RNA: Hepatitis C virus ribonucleic acid
HepMig: Study acronym for ‘Health care situation for hepatitis B and C in Germany among migrants from selected countries’
RKI: Robert Koch Institute, Berlin, Germany
