# Supplementary material for: Developing a tailored bio-behavioural survey on viral hepatitis among migrants: mixed-methods preparations for the HepMig pilot study, Germany, July 2022–March 2023
Source: BMC Res Notes. 2025 Oct 20;18:438. doi: 10.1186/s13104-025-07516-5 (PMC12539218; doi:10.1186/s13104-025-07516-5)
Supplement: Supplementary file 1 — Supplementary Material 1. [file 13104_2025_7516_MOESM1_ESM.docx]

## Supplementary material

**Supplement 1: Semi-structured interview guide**

1. Please describe your current activities / the work of your institution.

2. Where and how could people with a history of migration from Bulgaria, Romania, Russia, Syria, and Türkiye be reached for the study?

3. How could the potential study participants be approached?

4. Where and by whom could the data collection be carried out?

5. How could the data collection instruments be designed?

6. What need is there for translated study materials and language/cultural mediation?

7. Which other relevant discussion or cooperation partners, institutions or associations can you recommend for an exchange?

8. May we contact you again in the further course of the study planning?

9. Do you have any questions, additional suggestions or comments for us?

| **Number of residents with …** | **Berlin  (urban district)** | **Bremen  (urban district)** | **Dortmund  (urban district)** | **Duisburg  (urban district)** | **Essen  (urban district)** | **Frankfurt (Main)  (urban**  **district)** | **Hamburg  (urban district)** | **Cologne  (urban district)** | **Munich  (urban district)** | **Nuremberg  (urban district)** |
| --- | --- | --- | --- | --- | --- | --- | --- | --- | --- | --- |
| … Bulgarian citizenship | 32,095 | 6,480 | 4,490 | 14,035 | 2,780 | 9,040 | 8,835 | 8,480 | 12,765 | 6,150 |
| … Romanian citizenship | 27,155 | 2,790 | 5,630 | 9,225 | 5,160 | 11,620 | 10,005 | 5,325 | 18,170 | 15,035 |
| … Russian citizenship | 27,480 | 2,935 | 2,050 | 1,240 | 2,095 | 3,740 | 10,380 | 4,725 | 11,035 | 3,685 |
| … Syrian citizenship | 43,475 | 14,790 | 13,630 | 11,875 | 15,900 | 3,020 | 17,725 | 9,010 | 4,850 | 7,120 |
| … Turkish citizenship | 106,925 | 19,860 | 22,310 | 33,180 | 15,535 | 24,930 | 44,285 | 52,155 | 38,010 | 16,760 |
| … other non-German  Citizenships | 611,270 | 68,450 | 76,600 | 49,825 | 74,245 | 183,840 | 237,145 | 146,110 | 381,990 | 90,635 |
| … German and non-German  citizenships | 3,677,472 | 563,290 | 586,852 | 495,152 | 579,432 | 759,224 | 1,853,935 | 1,073,096 | 1,487,708 | 510,632 |

**Supplement 2: Number of residents with selected citizenships among all residents with German and non-German citizenships by major cities, Germany, 2021. From: DESTATIS, Central Foreigners’ Registry 2021 (19), and Data from the municipal directory – Independent cities and rural districts by area, population and population density 2021 (22)**
